# First Newborn Baby to Receive Experimental Therapies Survives Ebola Virus Disease

**DOI:** 10.1093/infdis/jiw493

**Published:** 2017-01-23

**Authors:** Jenny Dörnemann, Chiara Burzio, Axelle Ronsse, Armand Sprecher, Hilde De Clerck, Michel Van Herp, Marie-Claire Kolié, Vesselina Yosifiva, Severine Caluwaerts, Anita K. McElroy, Annick Antierens

**Affiliations:** 1 Médecins Sans Frontières, Brussels; 2 Institute of Tropical Medicine, Antwerp, Belgium; 3 Médecins Sans Frontières, Conakry, Guinea; 4 Centers for Disease Control and Prevention; 5 Emory University Division of Pediatric Infectious Disease, Atlanta, Georgia

**Keywords:** Ebola, ZMapp, GS-5734

## Abstract

A neonate born to an Ebola virus–positive woman was diagnosed with Ebola virus infection on her first day of life. The patient was treated with monoclonal antibodies (ZMapp), a buffy coat transfusion from an Ebola survivor, and the broad-spectrum antiviral GS-5734. On day 20, a venous blood specimen tested negative for Ebola virus by quantitative reverse-transcription polymerase chain reaction. The patient was discharged in good health on day 33 of life. Further follow-up consultations showed age-appropriate weight gain and neurodevelopment at the age of 12 months. This patient is the first neonate documented to have survived congenital infection with Ebola virus.

**(See the editorial commentary by Hayden, Friede, and Bausch on pages 167–70.)**

Whereas mortality of Ebola virus (EBOV) disease (EVD) is high in all age groups, it is highest in fetuses and neonates. Nearly all pregnancies of EBOV-infected women end with miscarriage or stillbirth [1]. Since the identification of EBOV, 15 neonates born alive to EBOV-infected mothers have been documented. All died; the longest documented survival duration was 19 days [2]. During the 2014–2016 outbreak, Médecins Sans Frontières treated at least 54 EBOV-infected women with pregnancies in the second and third trimester and recorded 35 second-trimester miscarriages and deliveries in Ebola treatment centers. The single baby born alive died 2 days after birth (unpublished data).

EBOV transmission likely occurs in utero, as samples from amniotic fluid, placentas, and fetuses have tested positive for EBOV, but probably also during delivery and breastfeeding [3]. Possible reasons for the high mortality among fetuses and neonates include the immune-privileged uterine compartment, causing a high intrauterine and fetal viral load, as well as the immaturity of the fetal and neonatal immune system. Neonates are known to have lower complement and neutrophil levels and reduced antibody-dependent cellular cytotoxicity capacity, compared with adults [4]. Additionally, neonates have immature adaptive responses, including reduced antigen-specific T-cell and antibody responses [5]. We describe the clinical course and management of EVD in a late premature neonate who is the first documented survivor of congenitally acquired EVD.

## CLINICAL COURSE AND MANAGEMENT

### History, Findings, and Evolution of the Mother

Our patient's mother, a 25-year-old previously healthy woman, was admitted to the Nongo Ebola treatment center in Conakry, Guinea, 3 days after onset of symptoms (hyperthermia, asthenia, and conjunctival injection). She reported a 28-week pregnancy and confirmed feeling fetal movements. Quantitative reverse-transcription polymerase chain reaction (qRT-PCR) assay of a blood specimen collected from the woman, measured with Cepheid's GeneXpert system, revealed a cycle threshold (Ct) of 18.8. Standard supportive therapy was started. Given the high mortality risk among pregnant women with EBOV infection, we initiated treatment with the antiviral agent favipiravir on the second hospitalization day, following the procedures and dosage used in the JIKI trial [6].

On her fifth hospitalization day, the woman lost blood and amniotic fluid per vaginam, went into labor, and gave birth to a live girl 2 hours later. Despite administration of oxytocin and misoprostol, she developed severe vaginal bleeding with signs of coagulopathy and died of hemorrhagic shock the same day.

### Initial Assessment of the Neonate

At delivery, we saw a viable female neonate with good primary adaptation. Gestational age was estimated to be 35–36 weeks. General and neurologic examination did not reveal pathologies. Birth weight was estimated to be 2800 g. qRT-PCR analysis of a capillary blood specimen obtained from the infant 45 minutes after birth was positive, with a Ct of 29.4. The patient remained in the high isolation unit, with standard EBOV infection control precautions. She received formula milk, routine newborn care, and a 5-day course of ampicillin as prophylactic therapy.

### Administration of ZMapp and Leukocyte Transfusion

Considering the 100% case-fatality rate of EBOV-infected neonates and the limited treatment options, we requested access to ZMapp for monitored emergency use. After receiving approval from the Guinean authorities, we obtained informed consent from the patient's father for treatment and corresponding monitoring and data sharing. Acetaminophen was given as premedication. We administered ZMapp by intravenous infusion with incremental infusion rates, following standard procedures, at a dose of 50 mg/kg on days 2, 5, and 8 of life. Infusions lasted 6–10 hours under continuous monitoring, and no adverse events were recorded.

**Figure 1.**
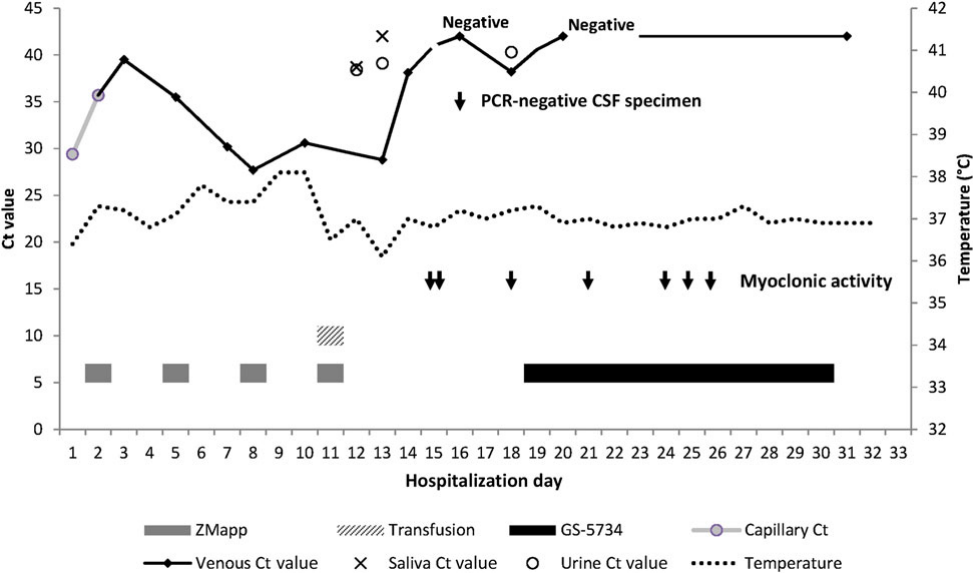
Time line of cycle threshold (Ct) values, body temperatures, and administration of therapeutic agents in a neonate with congenitally acquired Ebola virus disease. Abbreviations: CSF, cerebrospinal fluid; PCR, polymerase chain reaction.

Serial qRT-PCR analysis of venous blood samples showed an initial decrease of the viral load (ie, an increasing Ct) after the first infusion of ZMapp and a progressive increase (ie, a decreasing Ct) after the second and third ZMapp dose (Figure 1). ZMapp treatment did not result in suppression of viremia, unlike what has been observed in nonhuman primate (NHP) models following ZMapp treatment [7]. The monoclonal antibodies would be expected to neutralize circulating virus but, owing to the immature state of the patient's immune system, may not be able to stop ongoing intracellular virus replication, considering, for example, the relative deficiency of antibody-dependent cellular cytotoxicity and complement-mediated activity.

To support cell-mediated activity by the monoclonal antibodies, leukocyte transfusion from an EVD survivor was proposed. The Guinean National Transfusion Centre selected a voluntary EVD survivor with the same blood group and Rhesus factor as the neonate, to donate blood. The blood bag was hung for 4 hours to permit sedimentation, obtaining separation of erythrocytes, buffy coat, and plasma. Erythrocytes and plasma were removed, and 30 mL was extracted from the remaining buffy coat. This preparation would have contained mostly neutrophils but probably also contained mononuclear cells, such as lymphocytes, natural killer cells, and monocytes, although no cellular count was performed. On day 11 of life, we gave this preparation to the patient over 2 hours, followed 4 hours later by a fourth dose of 50 mg/kg ZMapp. No adverse events were recorded, despite the multiple risks associated with leukocyte transfusion [8].

### Further Development and Administration of GS-5734

Clinically, the patient remained in good condition. Fever, defined by axillary temperature of >37.5°C, was noted on days 6, 9, and 10 of life, with a maximum temperature of 38.1°C. To treat a possible concomitant bacterial infection, we started ceftriaxone treatment intravenously on day 7. Additionally, a course of artesunate-amodiaquine was administered as therapeutic coverage for possible malaria. Fever was treated with acetaminophen. The following days, high Ct values were found in saliva and urine specimens, while Ct values decreased in blood (Figure 1), suggested ongoing viral replication.

Fifteen days after birth, the patient developed clonic muscle activities in both arms and legs that lasted for approximately 2 minutes. A loading dose of 20 mg/kg phenobarbital was administered. A second episode occurred some hours later during sleep, involving only the left side and lasting for approximately 1 minute. A second dose of 10 mg/kg phenobarbital was given.

A lumbar puncture was performed on day 16. No EBOV presence could be detected in the cerebrospinal fluid (CSF). As there was visible blood in the CSF specimen, interpretation of findings of protein and cell analyses was not possible. Intravenous ampicillin was added to the ongoing ceftriaxone treatment to provide full antibiotic coverage for typical bacterial neonatal pathogens. Another episode of myoclonic activity, involving the left body side, occurred on day 18 and was treated with phenobarbital at a daily maintenance dose. Findings of qRT-PCR analysis of a venous specimen were negative on day 16 but positive on day 18, with a Ct of 38.2.

Interpretation of the exact nature of the intermittent myoclonic events was challenging in the absence of electroencephalography and medical imaging studies. While intermittent myoclonic events in neonates can be due to metabolic causes and drug side effects or be benign, they can also be a manifestation of central nervous system (CNS) pathology [9]. Even in the absence of detectable EBOV RNA in the CSF, viral replication in the CNS of the patient could not be excluded, based on the patient's low-level persistent viremia and on literature reports of EBOV isolation from immune-privileged sites even after negative results of venous qRT-PCR [10, 11]. Therefore, we considered the use of a small-molecule antiviral drug that had the potential to cross the blood-brain barrier to treat CNS EVD. Favipiravir is contraindicated in children aged <1 year because of the immaturity of the enzyme system responsible for the favipiravir metabolic pathway [12].

A novel broad-spectrum antiviral, the prodrug nucleotide analogue GS-5734 (Gilead Sciences), has been shown to inhibit EBOV replication with low cytotoxicity in multiple human cell types and has demonstrated 100% survival rates in EBOV-infected rhesus monkeys when given therapeutically up to 3 days after infection [13]. A 14-day course of GS-5734 was previously given in emergency use to an adult EVD survivor who relapsed with a severe meningo-encephalitis syndrome with no severe adverse events recorded [14].

Because of the invariably fatal outcome of congenitally infected neonates with EBOV, the suspicion of EBOV activity in the CNS, and the absence of proven treatment options, we proposed the monitored emergency use of GS-5734. After obtaining agreement from the manufacturer, approval from the national authorities and ethics committee, and informed consent from the father, we started treatment on day 19 of life. The compound was given at a daily dose of 10 mg, diluted in 50 mL of normal saline, by intravenous infusion over 2 hours under continuous monitoring. Dose determination was based on preclinical and clinical data indicating presumed efficacy for EVD treatment in humans, adapted to the age, weight, and kidney function of the patient [15]. Because nephrotoxicity was found in animal toxicology studies to be a known risk associated with GS-5734 use, creatinine levels (Table 1) and fluid balance were followed daily, along with other serum chemistry analyses.

**Table 1.** Laboratory Values During the Course of Illness

Variable	Day of Life																	
	14	15	16	17	18	19	20	21	22	23	24	25	26	27	28	29	30	31
Hemoglobin level, g/dL	16.7	13.9	…	…	14.6	12.6	…	11.9	12.2	11.6	…	…	…	…	…	11.2	…	…
Creatinine level, mg/dL	0.4	0.4	…	…	0.5	0.5	0.5	0.4	0.2	0.3	0.5	0.5	0.5	0.3	0.5	0.3	0.4	0.2
Urea level, mg/dL	3	3	…	…	<2	3	3	3	6	3	…	…	6	…	…	6	…	5
ALT level, U/L	23	20	…	…	15	20	17	19	18	10	…	…	21	…	…	20	…	20
AST level, U/L	49	47	…	…	37	33	32	38	27	29	…	…	44	…	…	34	…	35
Bilirubin level, mg/dL	0.5	0.6	…	…	0.5	0.4	0.4	0.5	0.5	0.4	…	…	0.4	…	…	0.4	…	0.4
CK level, U/L	101	158	…	…	…	…	178	103	142	223	…	…	260	…	…	232	…	230
Amylase level, U/L	19	12	…	…	10	11	6	14	12	18	…	…	10	…	…	7	…	7
CRP level, mg/L	<5	<5	…	…	6	<5	<5	<5	5.2	5.6	…	…	<5	…	…	<5	…	<5
Sodium level, mmol/L	134	133	…	…	130	131	129	130	130	132	…	…	129	…	…	131	…	131
Potassium level, mmol/L	5.9	4.7	…	…	5.8	4.5	5.1	5.5	4.3	5.5	…	…	5.1	…	…	4.5	…	5.1
Calcium level, mmol/L	10.7	10.3	…	…	10.1	9.1	9.5	10	9.5	9.9	…	…	9.9	…	…	9.6	…	10.3
Random blood sugar level, mg/dL	72	78	…	…	73	75	72	77	90	85	…	…	76	…	…	79	…	80

Abbreviations: ALT, alanine aminotransferase; AST, aspartate aminotransferase; CK, creatinine kinase; CRP, C-reactive protein.

Episodes of myoclonic activity involving different body parts and of variable duration occurred on days 21, 24, 25, and 26. These episodes, together with data from NHP studies to determine the optimal duration of the treatment, supported the decision to continue the full 12-day treatment regimen, despite the negative venous qRT-PCR results on the second day of GS-5734 initiation. The whole course of treatment with GS-5734 was well tolerated without any evidence of drug-related toxicity. The creatinine level remained normal, and urine output was adequate at all times.

### Discharge and Follow-up

Except for the episodes of myoclonic seizures, no signs of pathology appeared, and liver and kidney function remained normal. Feeding went well, and the body weight steadily increased. The patient was discharged on day 33 of life with a weight of 3100 g. Results of qRT-PCR for detection of EBOV in blood, urine, and skin swabs were negative at discharge. Weekly follow-up to the age of 12 months showed neurologic development according to age, normal growth (15th percentile for age), and the absence of pathology.

## DISCUSSION

We described a neonate who had congenital EBOV infection, received 3 different experimental therapies, and made a full clinical recovery. Several factors might have contributed to the favorable outcome. Viral transmission might have occurred late in the pregnancy and been limited during the brief labor and delivery, resulting in the neonate having a low viral load at birth. It cannot be excluded that the favipiravir given to the mother had an effect on the viral replication in the fetus.

A full course of ZMapp did not result in suppression of viremia; however, the monoclonal antibodies may have played a role in partial virologic control through neutralization of a proportion of circulating virus, whereas intracellular replication was likely not controlled, resulting in ongoing viremia. Following the combination of a survivor's leukocyte transfusion and a fourth ZMapp dose, Ct levels increased rapidly. As no data exist on EBOV Ct changes over time in neonates, it is unclear what impact these interventions had upon viremia in the patient.

GS-5734 had never been administered to a patient with a primary EBOV infection or to any pediatric patient. The indication was primarily to clear the virus suspected to be replicating in the CNS. As this could never be demonstrated, neither can the potential therapeutic effect of GS-5734.

It was feasible to administer these treatments by carefully monitored slow infusion rates. Simple point-of-care, field-based laboratory monitoring was used, owing to the constraints of infection control and lack of diagnostic options. Imaging services and microbiologic examinations were not possible in the given setting. Collection of blood samples of sufficient volume to enable testing of pharmacokinetics or antibody titers was impossible because of challenging venous access following multiple venous catheterizations. Performance of lumbar puncture in full personal protective equipment on a neonate was also challenging. We attempted to repeat the procedure to screen for EBOV and GS-5734 in CSF at the end of treatment, but after an unsuccessful attempt we decided it was unjustified to pursue this invasive procedure on a clinically well child.

Given the 100% case-fatality rate for congenitally acquired EVD, we found that an increased level of risk was acceptable and chose to administer experimental treatments that had not been given to patients in this age group and had an unproven effect in EVD. Further research will be needed to show whether the positive outcome is attributable to one or a combination of the administered treatments.
